# Imaging Findings of Primary Squamous Cell Carcinoma of the Liver: Case Presentation and Literature Review

**DOI:** 10.2174/0115734056344249250108062855

**Published:** 2025-03-17

**Authors:** Yichuan Mao, Xiuzhen Yao, Gui Xu, Feng Yang, Xiangqun Zhou, Xiaoqin Wu, Weiqun Ao, Jun Lin

**Affiliations:** 1 Department of Radiology, Tongde Hospital of Zhejiang Province, Hangzhou, Zhejiang Province, China; 2 Department of Ultrasound, Putuo People’s Hospital, School of Medicine, Tongji University, Shanghai, China; 3 Department of Ultrasound, Shangrao Guangxin District People's Hospital, Shangrao, China; 4 Department of Pathology, Shangrao Guangxin District People's Hospital, Shangrao, China; 5 Department of Medical Oncology, Shangrao Guangxin District People's Hospital, Shangrao, China; 6 Department of Radiology, Shangrao Guangxin District People's Hospital, Shangrao, China; 7 Department of Interventional Ultrasound, Shangrao Guangxin District People's Hospital, Shangrao, Jiangxi Province, China

**Keywords:** Primary squamous cell carcinoma, Liver, Ultrasound, Computed tomography, Case report, Squamous Cell Carcinoma, Alanine aminotransferase

## Abstract

**Introduction:**

Primary Squamous Cell Carcinoma of the Liver (PSCCL) is an exceptionally rare clinical entity characterized by diagnostic challenges, aggressive behavior, and poor prognosis. Globally, few studies have investigated PSCCL.

**Case Presentation:**

We report the case of a 76-year-old male patient with PSCCL, detailing his clinical presentation and imaging findings, to offer insights into the preoperative diagnosis of this disease. The patient presented with upper abdominal pain that had lasted for over two weeks without any specific triggers. Laboratory tests revealed abnormal liver function. Ultrasound examination showed a large, solid, hypoechoic mass in the right anterior lobe of the liver with heterogeneous internal echoes. Color Doppler imaging detected limited blood flow signals. Contrast-enhanced Computed Tomography (CT) of the whole abdomen revealed a low-density mass with indistinct margins in the right lobe of the liver, showing uneven and progressive peripheral enhancement. Comprehensive whole-body CT, gastroscopy, and liver biopsy were performed, excluding metastatic disease in other organs. Based on the pathological findings from a percutaneous ultrasound-guided liver biopsy, the patient was diagnosed with PSCCL.

**Conclusion:**

PSCCL is a rare malignancy that presents significant diagnostic difficulties, often evading easy identification through clinical and imaging examinations. This case report aims to contribute to improving the preoperative diagnosis of PSCCL.

## 1 INTRODUCTION

Squamous Cell Carcinoma (SCC) arises from the malignant transformation of squamous cells in organs lined by the squamous epithelium, such as the skin, lungs, esophagus, cervix, and vagina [1]. Given the lack of squamous epithelial tissue in the liver, Primary Squamous Cell Carcinoma of the Liver (PSCCL) is exceedingly rare, comprising only 0.2% of all primary liver cancers [2]. Diagnosing PSCCL based solely on clinical or imaging findings is challenging, as it can easily be mistaken for other types of liver cancer. In this report, we present the case of a man diagnosed with PSCCL confirmed through ultrasound and Computed Tomography (CT) examinations. Additionally, we review relevant literature to provide further insights. This case may offer a valuable reference for the diagnosis of PSCCL.

## 2 CASE PRESENTATION

A 76-year-old man presented to our hospital in June 2022 with epigastric pain that had persisted for over two weeks without any discernible triggers. The pain was described as paroxysmal. He denied symptoms of chills, fever, nausea, vomiting, chest tightness, or dyspnea.

On physical examination, the patient’s vital signs were as follows: body temperature: 36.3°C, pulse: 82 beats/min, respiration rate: 20 breaths/min, and blood pressure: 125/63 mmHg. Signs of chronic disease and significant weight loss (10 kg over the past two months) were noted. He was alert and oriented, with no jaundice observed in the skin or mucous membranes. His abdomen was soft and protuberant, with tenderness in the upper abdomen but no rebound pain, palpable masses, or hepatosplenomegaly. Murphy’s sign and shifting dullness were negative. The patient had no family history of genetic disorders or hepatitis virus infection and no prior history of surgery or other underlying conditions were recorded.

Laboratory tests revealed the following results: serum Alanine aminotransferase (ALT): 45.00 U/L, Aspartate aminotransferase (AST): 34.00 U/L, total bilirubin: 5.70 μmol/L, direct bilirubin: 1.90 μmol/L, indirect bilirubin: 3.80 μmol/L, total protein: 69.10 g/L, albumin: 33.10 g/L, hemoglobin: 109 g/L, and Cancer Antigen (CA): 19-9, 28.41 U/mL. No abnormalities were detected in other tumor markers. Coagulation parameters were within normal ranges, and the patient tested negative for hepatitis B, C, and E. Inflammatory markers were also normal.

**Fig. (1):**
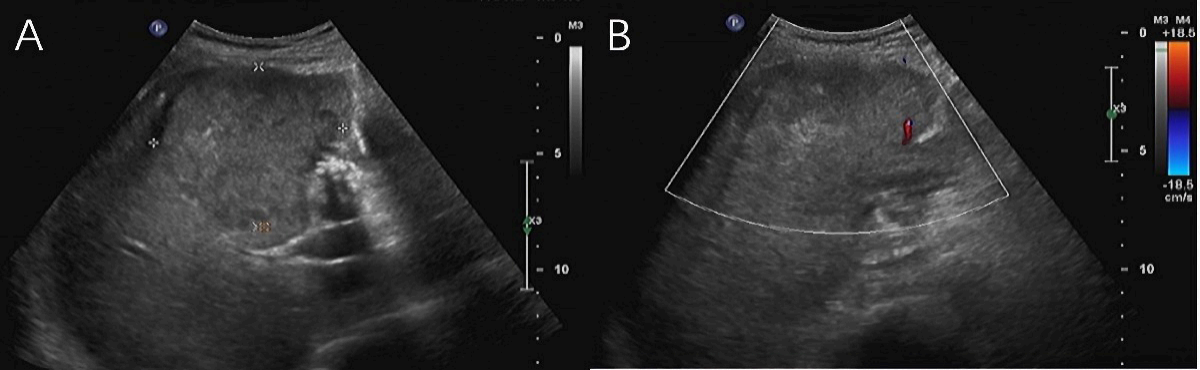
(**A**) Ultrasound imaging revealed a large, solid, hypoechoic mass with unclear margins and heterogeneous internal echo patterns located in the right anterior lobe of the liver. (**B**) Color Doppler imaging showed limited blood flow signals within the mass.

**Fig. (2):**
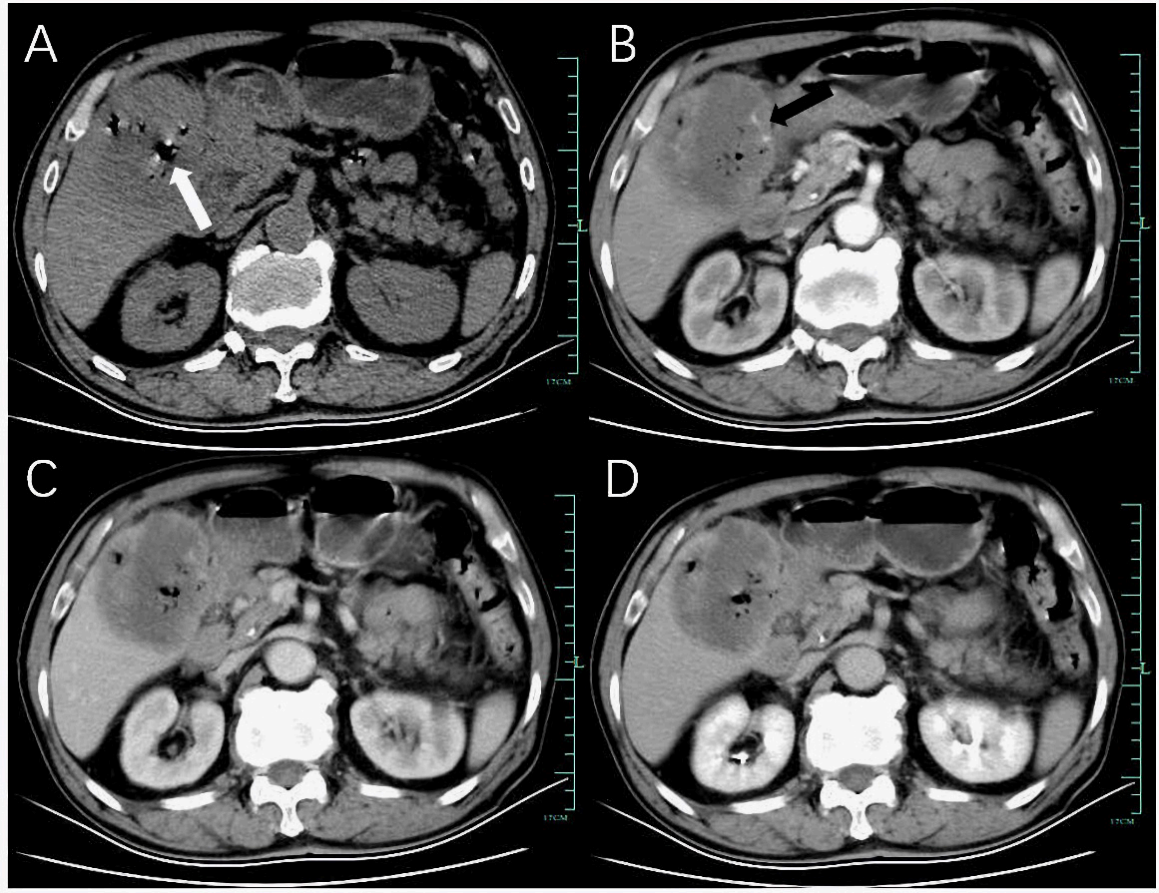
(**A**) Computed Tomography (CT) demonstrated a low-density mass in the right lobe of the liver, with post-puncture gas accumulation (white arrow). (**B**) In the arterial phase, the mass margins showed uneven enhancement, with small blood vessels originating from the hepatic artery noted (black arrow). (**C** and **D**) In the portal venous and delayed phases, the mass margins remained enhanced.

**Fig. (3):**
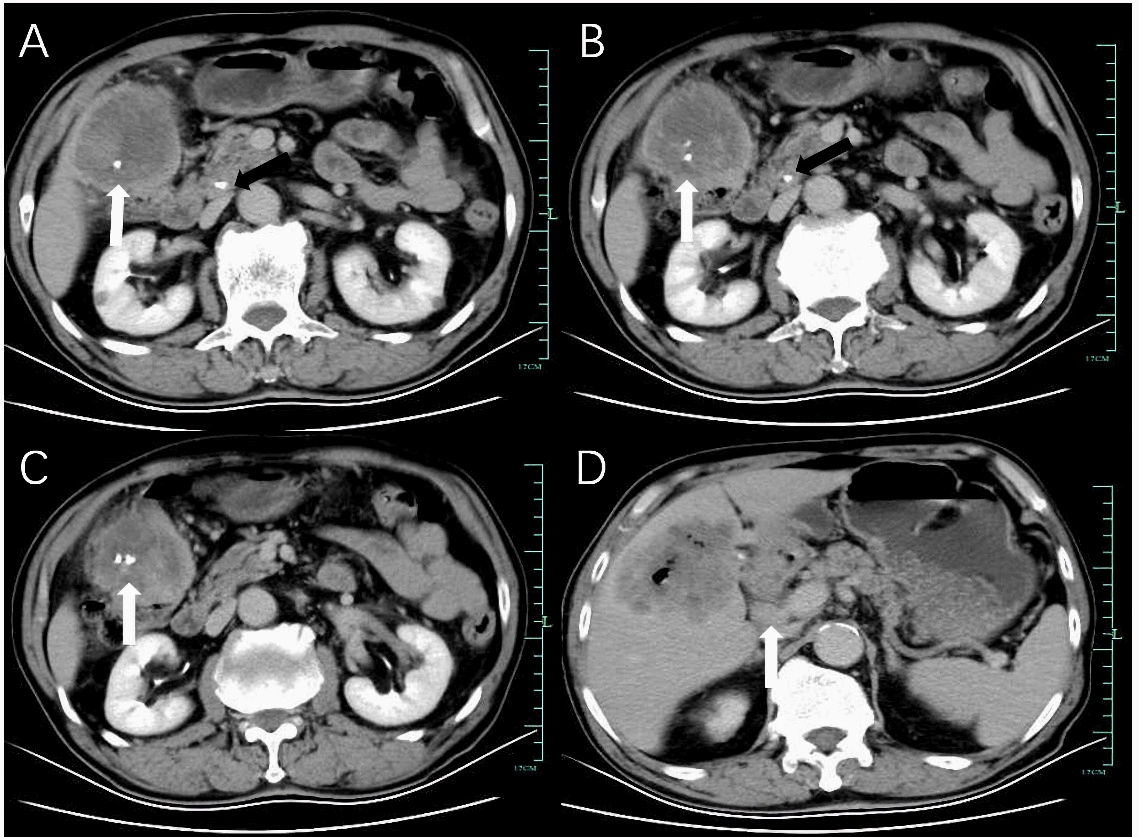
Observations during the equilibrium phase. (**A**–**C**) CT images revealed calcifications within the mass (white arrow) and common bile duct stones (black arrow). (**D**) Enlarged hilar lymph nodes were observed (white arrow).

Ultrasound examination revealed hepatomegaly with a slightly irregular shape but a smooth capsule. A large, solid hypoechoic mass measuring 73 mm × 69 mm with unclear margins and heterogeneous internal echoes was found in the right anterior lobe of the liver (Fig. **1A**). Color Doppler imaging demonstrated limited blood flow signals (Fig. **1B**). Contrast-enhanced Computed Tomography (CT) of the abdomen showed a low-density mass in the right lobe of the liver, measuring 74 mm × 68 mm × 57 mm, with indistinct margins and nodular calcifications. The mass exhibited uneven, progressive peripheral enhancement with non-enhancing necrotic areas and enhanced solid components. Arterial phase CT revealed small vessels supplying blood from the hepatic artery. Scattered gas was noted within the mass post-biopsy (Fig. **2**). Additionally, stones were identified in the lower segment of the common bile duct, and multiple enlarged lymph nodes were detected in the liver hilum and retroperitoneal region (Fig. **3**). Initially, the patient was misdiagnosed with intrahepatic cholangiocarcinoma due to the tumor’s invasive nature and the absence of cirrhosis.

Further evaluations included head and neck CT, which showed no abnormalities, and chest CT, which revealed scattered chronic infection foci in both lungs. Endoscopy identified a stage A duodenal bulb ulcer and chronic atrophic gastritis with erosion.

A percutaneous ultrasound-guided liver biopsy was performed after obtaining informed consent. The patient assumed the right anterior oblique position for the procedure. Under ultrasound guidance, a biopsy site was selected on the right intercostal surface. After skin disinfection, draping, and local anesthesia with 2% lidocaine, an 18-G, 100-mm puncture gun was used to obtain three tissue samples (5–12 mm in length). The biopsy site was pressed and bandaged, with no active bleeding. The tissue samples were sent for pathology screening, and the patient returned to the ward. He remained in bed for 24 hours, during which his vital signs were stable, and no complications occurred.

**Fig. (4):**
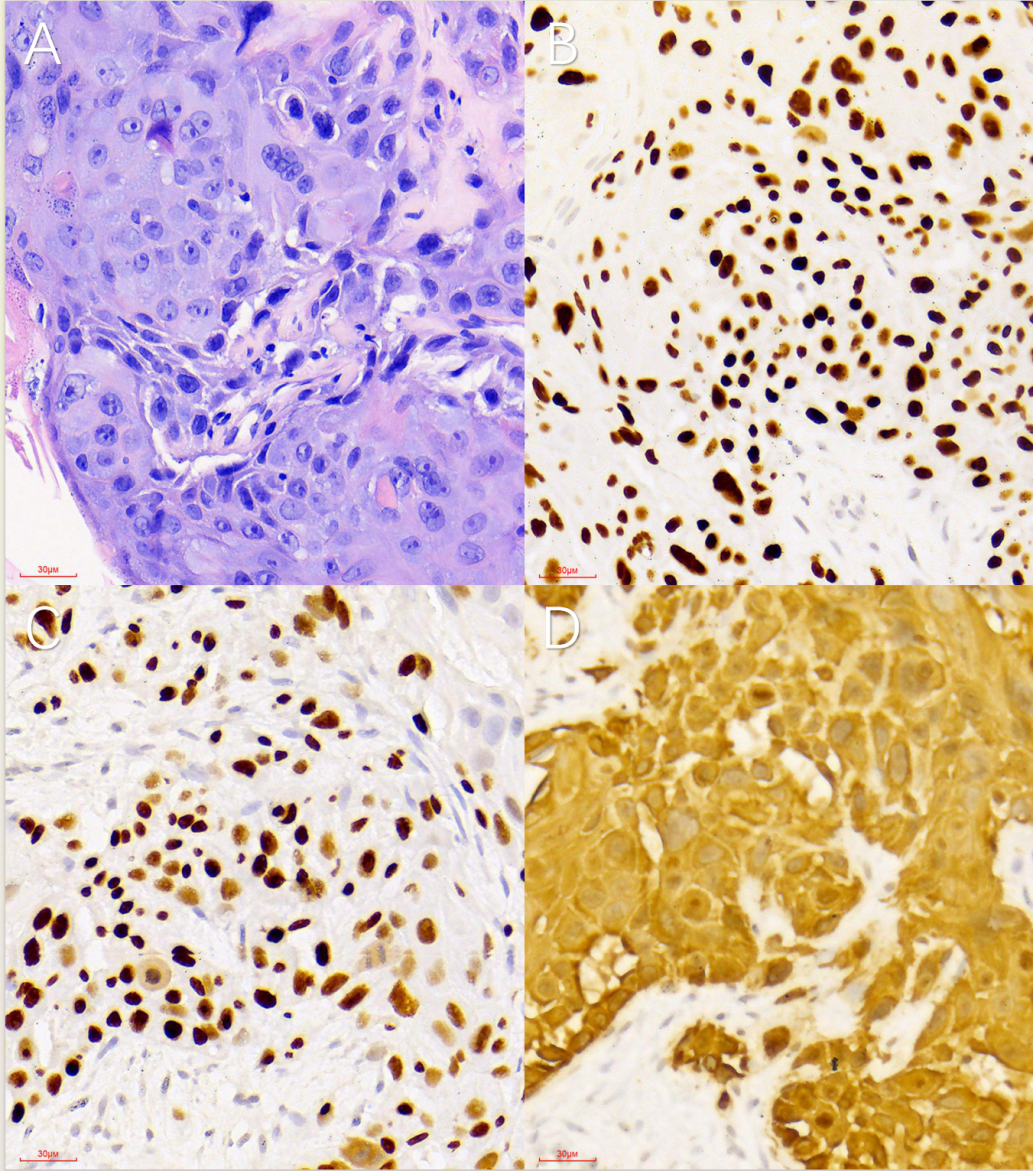
Immunohistochemical images. (**A**) Hematoxylin and eosin staining (40x) showed tumor cells with infiltrative growth, arranged in nests or sheets, with fibrous tissue proliferation at the periphery. Cells exhibited abundant cytoplasm, irregular karyotypes, and large, deeply stained nuclei. (**B**–**D**) Immunohistochemical staining showed positive results for P40 (B; 40x), P63 (C; 40x), and cytokeratin 5/6 (D; 40x).

Microscopic examination of the biopsy samples revealed infiltrative tumor cells arranged in nests and sheets (Fig. (4A), with fibrous tissue proliferation at the periphery. The tumor cells exhibited abundant cytoplasm, irregular nuclei, and hyperchromatic features. Immunohistochemical analysis (Fig. (4B–D) showed positivity for Ki-67 (40%), P40 (+), P63 (+), and cytokeratin 5/6 (+), with negative results for alpha-fetoprotein, hepatocyte, cytokeratin 19, cytokeratin 7, and thyroid transcription factor 1. These findings confirmed the diagnosis of Squamous Cell Carcinoma (SCC).

No clinical or imaging evidence of extrahepatic tumors was identified, and the patient had no history of SCC in the digestive, respiratory, or urinary systems. Therefore, based on pathological and immunohistochemical findings, a diagnosis of Primary Squamous Cell Carcinoma of the Liver (PSCCL) was made.

During hospitalization, the patient received symptomatic treatment, including gastric and liver protection, anti-infection therapy, analgesia, and nutritional support. After his condition stabilized, the patient opted to discontinue surgical treatment and was followed up in the outpatient department. However, at discharge, laboratory tests indicated abnormal liver function, with the following results: ALT: 393.00 U/L, AST: 520.00 U/L, total bilirubin: 316.87 μmol/L, direct bilirubin: 216.88 μmol/L, indirect bilirubin: 99.99 μmol/L, total protein: 55.30 g/L, albumin: 23.10 g/L, globulin: 32.20 g/L, albumin/globulin ratio: 0.7, pyruvic acid: 212 μmol/L, and glutathione reductase: 220 U/L. Tragically, the patient succumbed to liver failure six months after the diagnosis in December 2022.

## 3 DISCUSSION

Liver SCC typically metastasizes from primary sites, such as the lungs and gastrointestinal tract. Therefore, PSCCL is diagnosed only after ruling out metastatic tumors. The causes and pathogenesis of PSCCL remain unclear. Studies have suggested associations between PSCCL and conditions such as intrahepatic bile duct stones, liver teratomas, liver cysts, Caroli’s disease, and cirrhosis [3-13]. Squamous metaplasia induced by chronic inflammation may lead to cancer. Our patient had common bile duct stones, and the calcification within the mass might have originated from intrahepatic bile duct stones. PSCCL may also occur in the absence of any of the aforementioned conditions [2, 14, 15]. This could be due to pluripotent stem cells in the liver transforming into malignant squamous cells, hepatocytes, or bile duct epithelial cells under the influence of various carcinogenic factors, thereby leading to SCC.

Most patients with PSCCL experience liver discomfort, abdominal bloating, and anorexia. Systemic symptoms, such as epigastric discomfort, pain, gastrointestinal issues, fever, anemia, fatigue, or weight loss, may manifest depending on the tumor's location, growth duration, and size [2, 16]. These symptoms are non-specific, making it challenging for clinicians to differentiate PSCCL from other liver diseases. Our patient primarily presented with upper abdominal pain and a lean face, symptoms that are non-specific but common in liver diseases.

Laboratory evidence suggests that some patients have elevated levels of ALT, AST, and bilirubin, most patients have normal levels of alpha-fetoprotein, and many patients have elevated levels of CA19-9, CA 12-5, carcinoembryonic antigen, and SCC-related antigens [1, 2]. Our patient presented with only a slightly elevated CA19-9 level, while other tumor markers remained normal. However, no specific serum biomarkers for PSCCL have been identified, complicating the diagnostic process.

The global incidence of PSCCL is extremely low. Due to the limited clinical findings on PSCCL, it is often misdiagnosed as another type of liver cancer. CT is the predominant imaging modality for the preoperative diagnosis of PSCCL [2]. A PubMed search revealed that very few imaging studies have been conducted on PSCCL, with most relying on CT and some on MRI. Although PSCCL lacks distinctive imaging features, a multimodal imaging approach can enhance diagnostic accuracy [17]. The imaging data presented here are comprehensive, including findings from ultrasound and CT examinations. Based on this case and a review of the literature, we summarized the imaging features of PSCCL (Table **1**).

Ultrasound is typically used as an initial screening tool. PSCCL is characterized by the presence of single or multiple, round or lobulated, hypoechoic or hyperechoic, and cystic or solid hepatic masses with unclear margins. The tumor wall exhibits uneven thickness and echo patterns, which may be accompanied by intrahepatic or extrahepatic bile duct dilation. Blood flow may be visible in and around the mass. CT scans, one of the primary diagnostic tools for PSCCL, typically reveal single or multiple, round or lobulated hepatic masses with unclear margins. Plain CT scans show the tumor as a low-density region, with necrotic areas of even lower density at the center. Contrast-enhanced CT reveals mildly to moderately enhanced mass margins and parenchymal cells during the arterial phase, with sustained enhancement in the portal venous and delayed phases. Occasionally, intrahepatic bile duct stones, intrahepatic bile duct dilatation, liver cysts, lymph node metastasis and adjacent tissue invasion can be observed [18-22]. Our patient had a low-density mass in the right liver lobe with unclear margins and multiple gas-density shadows, likely due to a liver biopsy performed before the CT examination. On contrast-enhanced CT, the mass margins and parenchymal cells exhibited uneven and progressive enhancement, with no enhancing necrotic areas within the mass. These findings were similar to those of other liver cancers, leading to an initial misdiagnosis of intrahepatic cholangiocarcinoma.

The diagnosis of PSCCL relies on pathological examination. Microscopic findings show that SCC is characterized by varying cell sizes, nested structures, visible intercellular bridges, large and deeply stained nuclei, atypia, mitotic phases, abundant cytoplasm, bichromophilia, parakeratosis, and keratinized beads. In our patient, no ductal carcinoma tissue was observed. Some studies suggest that positive expression of P40, P63, and CK5/6 can serve as diagnostic markers for PSCCL [21, 23]. CK19 and CK7 can be used to mark the structure of glandular ducts, which can be used to differentiate primary SCC from the neoplastic cells of the bile ductular ontogeny. The absence of TTF-1 expression may rule out the possibility of metastasis from small-cell lung cancer or thyroid cancer to the liver. The microscopic and immunohistochemical findings in this case were consistent with the typical manifestations of SCC.

Given the rarity of liver primary squamous carcinoma, no standardized therapeutic regimen exists. Reported treatments include surgical resection, chemotherapy, radiotherapy, Transcatheter Arterial Chemoembolization (TACE), or combinations of these therapies, being similar to treatments for Hepatocellular Carcinoma (HCC). However, PSCCL and HCC are malignancies with different origins, and PSCCL tends to exhibit more aggressive characteristics, leading to a generally poor prognosis. Survival is generally no longer than 1 year [2, 16], much lower than that of HCC patients [24]. In previously reported cases, patients who underwent radical surgery had longer overall survival compared to those receiving palliative care [1, 2]. Boscolo and Lee *et al*. [25, 26] reported that intrahepatic arterial chemotherapy with cisplatin and 5-fluorouracil showed some efficacy. Although early intervention may extend survival, the median overall survival and disease-specific survival were 7.7 months (range 0.0–76.0 months) and 2.0 months (range 0.0–20.0 months), respectively [1]. Recent studies on HCC have linked differential gene expression, immune infiltration levels, survival outcomes, and clinical phenotypes in HCC patients, contributing to advances in prognosis research and immunotherapy [27]. However, research on the treatment and prognosis of PSCCL remains limited. Further studies and an accumulation of case data are essential for developing more effective treatment strategies to enhance patient’s quality of life and outcomes. Our patient’s overall survival was consistent with previous reports.

## CONCLUSION

In conclusion, PSCCL is an extremely rare and highly aggressive hepatic malignancy characterized by non-specific clinical and imaging features, making diagnosis and treatment challenging. This case report highlights the pathogenesis, clinical presentation, imaging characteristics, and prognosis of a patient with PSCCL, aiming to improve awareness and understanding of this rare malignancy among clinicians and radiologists.

## Figures and Tables

**Table 1:** Findings of imaging examinations for primary squamous cell carcinoma of the liver.

**Imaging Examination**	**Imaging Findings**
Ultrasound examination	An irregular, hypoechoic mass with heterogeneous echotexture, accompanied by peripheral bile duct dilatation in some cases, may demonstrate blood flow within and surrounding the mass.^14,18,19^
Computed tomography	Single or multiple low-density masses showing mild delayed peripheral enhancement with central necrosis; some masses are associated with air-fluid levels in the necrotic core, dilation of secondary biliary ducts with intraductal lithiasis, tumor thrombi in the middle hepatic vein, or multiple metastatic lymph nodes^2,3,15,19–22^
Magnetic resonance imaging	A large mass exhibiting prolonged T1WI and T2WI signals, high signal intensity on diffusion-weighted imaging (DWI), and low signal on the apparent diffusion coefficient (ADC), with some cases accompanied by peripheral bile duct dilation^1,19^

## Data Availability

The data and supportive information are available within the article.

## LIST OF ABBREVIATIONS

PSCCL: Primary squamous cell carcinoma of the liver
SCC: Squamous cell carcinoma
CT: Computed tomography
ALT: Alanine aminotransferase
AST: Aspartate aminotransferase
CA: Cancer antigen
WI: Weighted image
CK: Cytokeratin
MRI: Magnetic resonance imaging
TACE: Transcatheter arterial chemoembolization
HCC: Hepatocellular carcinoma

## STANDARDS OF REPORTING

CARE guidelines were followed.
